# Early Life Sleep Deprivation and Brain Development: Insights From Human and Animal Studies

**DOI:** 10.3389/fnins.2022.833786

**Published:** 2022-05-03

**Authors:** Ghalya Alrousan, Arham Hassan, Aditya Anilkumar Pillai, Fatin Atrooz, Samina Salim

**Affiliations:** Department of Pharmacological and Pharmaceutical Sciences, College of Pharmacy, University of Houston, Houston, TX, United States

**Keywords:** sleep deprivation, brain development, anxiety, depression, cognition

## Abstract

Adequate sleep especially during developmental stages of life, is considered essential for normal brain development and believed to play an important role in promoting healthy cognitive and psychosocial development, while persistent sleep disturbances and/or sleep deprivation during early life are believed to trigger many mental ailments such as anxiety disorders, depression, and cognitive impairment. Initially it was suggested that adverse mental health conditions adversely affect sleep, however, it is now accepted that this association is bidirectional. In fact, sleep disturbances are listed as a symptom of many mental health disorders. Of special interest is the association between early life sleep deprivation and its negative mental health outcomes. Studies have linked persistent early life sleep deprivation with later life behavioral and cognitive disturbances. Neurobiological underpinnings responsible for the negative outcomes of early life sleep deprivation are not understood. This is a significant barrier for early therapeutic and/or behavioral intervention, which can be feasible only if biological underpinnings are well-understood. Animal studies have provided useful insights in this area. This article focusses on the knowledge gained from the research conducted in the area of early life sleep deprivation, brain development, and behavioral function studies.

## Introduction

A question often arises during discussions of sleep, “How much sleep is good sleep?” The National Sleep Foundation (NSF) and the American Academy of Sleep Medicine (AASP)/Sleep Research Society (SRS) both have offered their recommendations. For example, infants between the ages of 4–11 months are recommended 12–15 h of sleep by NSF while AASP/SRS recommends 12–16 h of sleep. Children between the age of 6–13 years are recommended 9–11 h by NSF and children from ages 6–12 years are recommended 9–12 h. Teenagers are recommended 8–10 h by both organizations. NSF considers teenagers to be 14–17 year old while AASP/SRS classifies them as between 13 and 17 year old (Hirshkowitz et al., 2015). Clearly, adequate sleep during early life is essential for normal developmental processes. In fact, sleep is one of the major activities of the brain during early developmental phase of life, hence sleep reportedly plays an important role in healthy cognitive and psychosocial development during formative years of early life. Several studies have highlighted the important role sleep during early life period, plays in regulation of learning and memory functions (Jiang, 2019). For example, Seehagen et al. (2015) reported that an extended nap time within 4 h of learning a set of object pairings from toys allowed infants in the 6–12-month age range to retain newer behaviors better than having a 4- or 24 h delays, supporting the hypothesis that frequent napping for infants and small children leads to establishing better long-term memory. Important association between night-time sleep and memory also has been reported in other studies (Konrad and Seehagen, 2021; Seehagen et al., 2021). A study examining the relationship between sleep patterns and depressive symptoms, self-esteem, and grades of 2,259 Chicago students, aged 11–14 years, revealed that students who slept less in the sixth grade exhibited lower self-esteem and reported higher depressive symptoms (Fredriksen et al., 2004). In another study conducted on 135 healthy school children (69 boys and 66 girls), from second, fourth, sixth grades, sleep was monitored using actigraphy for 5 consecutive nights. Using a computerized neurobehavioral evaluation system, the neurobehavioral functioning (NBF) was assessed, and significant correlations were observed between sleep-quality and NBF measures, especially in young children. Children who obtained fragmented sleep exhibited lower performance on NBF measures, particularly on measures associated with complicated and tenuous continuous performance test (Sadeh et al., 2002). In another study, sleep problems in 3rd grade children were reportedly predicted intellectual stagnation, while longer sleep predicted better development of reading skills. Persistent problems falling asleep or maintaining sleep during childhood also predicted poorer scores due to the worsening of the children's executive functioning, but non-verbal reasoning, vocabulary, or fine motor skills are not greatly impacted (O'Brien and Mindell, 2005). According to a review conducted by Beebe et al. (2010), adolescent risk behaviors including cigarette smoking, alcohol consumption, illicit drug use, aggression, and suicidal tendencies, were reportedly associated with sleep disruption. Poor sleep patterns also are associated with daytime sleepiness, high stress, depressed mood, weight gain and obesity, alcohol use, caffeine intake, and cigarette smoking. Other studies have suggested that children whose average sleep duration, as measured via actigraphy, was short (<7.7 h), demonstrated a higher hyperactivity/impulsivity score as well as a higher attention-deficit/hyperactivity disorder total score but a similar inattention score compared with children sleeping more than 9.4 h (Paavonen et al., 2009). Furthermore, inadequate sleep has been linked to poor school performance (Dewald et al., 2010; Astill et al., 2012), and an increase in behavioral problems such as attention deficit, and emotional conditions such as anxiety and depression (Gregory and Sadeh, 2012; Maski and Kothare, 2013). In a longitudinal study, shorter sleep duration in children between 2.5 and 6 years old age range was associated with high levels of hyperactivity and engagement in disruptive behavior at school (Touchette et al., 2008, 2009). Early life sleep disturbances are also associated with later onset of anxiety and depression as a childhood and adolescent (Gessa et al., 1995; Gregory et al., 2005). The longitudinal studies suggest that early life sleep deprivation highly influences future affective and behavioral problems (Maski and Kothare, 2013).

Previously, both animal and human studies have suggested an important role of sleep deprivation in development of pathological anxiety. Persistent insomnia is reported to be associated with an increased risk of developing anxiety disorder (Alkadhi et al., 2013). In a rat model of sleep deprivation, 24 h sleep deprivation increased anxiety-like behavior of rats when assessed using open-field activity and light-dark exploration tests immediately following 24 h of sleep deprivation (Vollert et al., 2011). Levels of serum corticosterone, often associated with anxiety, also increased with sleep deprivation. Furthermore, sleep deprivation also increased oxidative stress markers in the cortex, amygdala, and the hippocampus, including 8-isoprostane and malondialdehyde (MDA) levels in the serum, suggesting involvement of oxidative stress mechanisms in regulation of sleep deprivation-related behavioral impairments.

Chronic sleep deprivation is reportedly linked to depression. The relationship between sleep and depression is believed to be associated through the serotoninergic system, which is active during wakefulness and inactive during sleep (Al-Abri, 2015). Moreover, serotonin release is significantly inhibited during slow-wave sleep and REM sleep. In an animal model of sleep deprivation, rats displayed depression-like behavior, metabolic, and microbial changes after chronic sleep deprivation for 7 days (Ma et al., 2019). This study found elevated levels of inflammatory cytokines, including IL-6, TNF-alpha, and CRP, and hyper-activated hypothalamic-pituitary-adrenal (HPA) axis in sleep deprived rats, which were postulated to play an important role in occurrence of behavioral deficits. Thus, there is enough support of the hypothesis that poor sleep during early life can have detrimental consequences on later life behaviors, cognitive and intellectual functions, and there is substantial evidence to suggest that sleep problems during early life may lead to social, cognitive and intellectual impairments. The mechanistic underpinnings remain unclear.

How sleep at early life modulates neurobehavioral functioning and cognition is not yet understood. Sleep spindles and slow wave activity, processes known to be affected through childhood and adolescence, is suggested to play an important role in regulation of neurobehavioral functions and growth, development and refinement of executive functions (Lopez et al., 2010; Maski and Kothare, 2013; Kurth et al., 2015). However, further research is needed to investigate the association between sleep and neurobehavioral development. Animal studies have offered valuable insights. This article will first review the normal sleep characteristics, followed by a review of preclinical studies focused on the association between sleep, early brain development and mental health, especially memory functions and emotional control.

### Sleep Physiology

Sleep is a physiological state characterized by reduced responsiveness to environmental stimuli. In vertebrates, sleep is considered as a specific pattern of synchronous electrical activity confined within the cortical area of the brain. The different stages of sleep are defined based on polysomnography (PSG) data. PSG involves simultaneous recording of the activity from the cortical area of the brain using electroencephalogram (EEG), eye movement via electrooculogram (EOG), and electromyogram (EMG)-mediated recording of the skeletal muscle activity. In humans and other mammals, sleep consists of two main phases: rapid eye movement (REM) sleep and non-REM (NREM) sleep. NREM sleep, also known as slow wave sleep, is characterized by high amplitude low frequency EEG waves, decreased muscle tone, and slow eye movement. While REM sleep, known as active sleep, is characterized by low amplitude high-frequency waves, muscle atonia, and rapid eye movement. EEG slow wave activity (delta power) in NREM sleep is considered as a marker of sleep propensity. In humans, NREM sleep is further classified into N1, N2, and N3 stages. Each NREM stage has a well-defined EEG wave pattern. During sleep, the different phases of sleep alternate in a tightly regulated pattern resulting in creation of sleep cycles (Stenberg and Porkka-Heiskanen, 1990). Sleep/wakefulness state is regulated by the interaction of two processes; the sleep homeostasis (process S) and the circadian system (process C) (Borbely, 1982a,b). Process C is regulated by the suprachiasmatic nucleus (SCN) of the hypothalamus. The SCN functions as the neural pacemaker of the circadian timing cycle. This pacemaker is autonomous and is essentially synchronized to environmental light/dark cycle and projects signals that control the timing and the execution of physiological functions including the sleep/wake cycle. The daily circadian rhythm is an internally driven process comprising of a 24-h cycle. A normal light/dark cycle includes a well-defined rhythm in which sleep onset and wakefulness onset occur at the same time every day. Sleep homeostasis or process S is believed to be regulated by generation of sleep pressure, which is initiated after a period of wakefulness. Thus, process S is believed to increase with extended wakefulness and known to decrease following sleep onset. Two hypotheses have been proposed in favor of the theory of generation of sleep pressure; *first*, is the involvement of the accumulation of sleep-promoting substances such as nitric oxide and adenosine, second is the role of resources of the restoration of energy (Abrahamson and Moore, 2006). The interaction of the sleep homeostatic mechanisms and the circadian rhythm regulates important elements of sleep, i.e., sleep timing, depth of sleep, and the duration of staying asleep. The interplay between the two processes is known to play a crucial role in regulation of functions related to body metabolism and maintenance of normal brain function and disruption in this interplay is responsible for contributing to many metabolic, psychiatric, and neurodegenerative diseases (Wulff et al., 2010).

Wakefulness is dependent on the ascending reticular activating system (ARAS), coordinated by several defined nuclei in the pons and midbrain areas. Cells promoting wakefulness are; noradrenergic (NE) cells of the locus coeruleus, serotonergic (5-HT) cells of the raphe nuclei, cholinergic (Ach) cells of the pedunculopontine tegmentum and laterodorsal tegmentum, glutamatergic cells (Glu) of the midbrain, and dopaminergic (DA) cells of the substantia nigra and ventral tegmental area. Projections of wake-promoting cells activate thalamocortical, hypothalamo-cortical, and cortico-basal systems. Furthermore, there are 5 other groups of wake-promoting cells including; (1) the histaminergic cells of the posterior hypothalamic area, (2) the hypocretin/orexin cells of the lateral hypothalamus, (3) the cholinergic cells of the basal forebrain, (4) the neuropeptide Y (NPY) containing cells within the suprachiasmatic nucleus, and (5) the glutamatergic neurons present in the ventro medial prefrontal cortex (vmPFC). Activation of these systems maintains stable wakefulness by cortical activation and inhibition of activity in sleep-promoting areas of the brain. However, cell lesions within a particular wake-promoting group of cells does not by itself induce changes in the amount of wakefulness. This raises the interesting possibility that different brain regions collectively contribute to the promotion and maintenance of wakefulness, but none by itself are required for generation of wakefulness. Sleep physiology especially the understanding of the initiation and maintenance of sleep has been a challenge for decades and more research is needed. Initiation of sleep is suggested to be a result of successive passive and active physiological processes which leads to blockage of cortical sensory gates (Datta, 2010). The passive mechanism which contributes to sleep initiation is a result of the generation and the accumulation of neuronal activity-dependent metabolites, which suppresses wakefulness and increases sleep inertia. Metabolic products produced during wakefulness, such as adenosine, nitric oxide, cytokines, and prostaglandins, accumulate and slow down wake-prompting neuronal activity, ultimately leading to slow down the production of metabolites. The active process of sleep initiation is represented by reducing the activity of wake-promoting neurons. Regulation of metabolite-dependent neuronal activity contributes to a state of a metabolite homeostasis (Datta, 2010). Data reported in various electrophysiological studies have suggested that the thalamic reticular nucleus seems to function as a pacemaker of sleep spindles generation (Steriade et al., 1993). The thalamic reticular nucleus serves as the sensory and internal signal operating at the gateway toward the cerebral cortex brain region and consists of exclusively the GABAergic neurons. The thalamic reticular nucleus contains two types of neurons; the thalamocortical relay neurons which are responsible for transmitting incoming sensory signals to the cerebral cortex, and the thalamo-reticular neurons which prevents transmission of sensory signals to the cerebral cortex. During wakefulness, the activity of the thalamo-cortical relay neurons is proportional to the activity of wake-promoting noradrenergic, serotonergic, and cholinergic cells. During this time the thalamic-reticular cells remain inactive. During the sleep initiation process, reduction in wake-promoting cells is believed to lead to reduction in activity of thalamocortical relay and the enhancement of thalamic-reticular cellular activity. Excitation of thalamic-reticular is responsible for further inhibiting thalamocortical relay by activation of the inhibitory postsynaptic GABA-B receptors (Steriade et al., 1993).

Consequently, cerebral cortex sensory signals at the cortical gate are blocked within the thalamus, depriving the cerebral cortex of receiving external information, as evident from high voltage and slow brain waves during NREM sleep, which is an indication of cortical inactivity (Stenberg and Porkka-Heiskanen, 1990; Kalia, 2006; Datta, 2010). Sleep is a common act engaged by all organisms during which the network of the neuronal/glial cells play an important role. Consequently, sleep becomes an important property of this neuronal network. Several theories have been proposed over the years to explain the function of sleep in terms of the properties of energy restoration, thermoregulation, waste elimination, and neuronal connectivity and plasticity augmentation, yet, sleep remains a challenging research area for scientists to explore and to promote greater understanding in this area (Krueger and Obal, 2003; Krueger et al., 2016).

## Sleep and Postnatal Brain Development

The process of brain development is initiated prenatally and continues throughout the postnatal period. Postnatal brain development is characterized by two major events; the cortical gray matter expansion or regression and development of white matter. Gray matter maturation is indicated by an increase in synapse formation, a process termed as synaptogenesis. The process of synaptogenesis is believed to be at its peak level during the childhood phase typically lasting between 2 and 3 years old. This phase of high synaptogenesis is followed by a period of rapid elimination and refinement of the presence of excess synapses, a process described as synaptic pruning. The synaptic pruning process continues until the adolescent stage of life. White matter development is indicated by neuronal myelination which occur during early life stages of life including the childhood and adolescent phase. The process of neuronal myelination continues until the early adult stage of development, and both processes of synaptogenesis and myelination are considered critical for optimization of neuronal connectivity and maintenance of normal neurobehavioral functions (Semple et al., 2013). Interestingly, remarkable changes reportedly take place in the sleep architecture during development, which parallels postnatal brain maturation time line, which indicates an important relationship between postnatal brain development and sleep health (Semple et al., 2013; Kurth et al., 2015). REM sleep is believed to be greatly concentrated in newborns comprising of ~50% of the total sleep that the infants get. During the first 2 years of life, REM sleep reduces to ~20–25% of total time spent sleeping, a pattern which is maintained throughout the developmental course (Louis et al., 1997). REM sleep reportedly promotes cortical plasticity which is important for consolidation of waking experiences in the developing brain (Dumoulin Bridi et al., 2015). And, localization, distribution, and coherence of sleep EEG captured from early childhood to late adolescence period, is reflective of the brain maturation processes. Cortical maturation follows a posterior to anterior path, similarly, SWA follows a posterior to anterior path within the brain regions with respect to brain maturation (Kurth et al., 2010b). Also, EEG activity, which measures the functional connectivity is strengthened by neuronal myelination, and increases with maturation in a region-specific manner (Tarokh et al., 2010; Kurth et al., 2013). Myelination is important for maintenance of brain connectivity and regulation of functional network maturation. Oligodendrocytes surround the axons with myelin sheaths offering an insulation of multiple layers which increases the conduction of action potential by 1,000-folds. Neuronal myelination is indicated by an increase in the volume of brain white matter. Neuronal myelination is believed to initiate prenatally and continues throughout the early adult life of humans. In rodents however, neuronal myelination reportedly continues until at least between the postnatal day (PND) 30–40. It is reported that oligodendrocyte proliferation and myelin synthesis occurs preferentially during sleep (Toth and Neumann, 2013). This information suggests that neuronal activity during sleep, may play an important role in brain development. By inducing neuronal myelination, neuronal activity is reported to enhance functional connections during brain development (Kurth et al., 2013, 2015). Thus, sleep is considered essential for proper neuronal network connectivity and brain maturation process to occur during the developmental period, a sensitive period of childhood.

Another important process during postnatal brain development is synaptogenesis. Synaptogenesis is the process of formation of new synapses between neurons. During brain development, synapse overproduction occurs, which is followed by synapse pruning and synapse elimination events. These processes highlight the complexity of neuronal circuits during the early life period of childhood and adolescence. It is during these critical periods of life that synaptic plasticity and cortical maturation are induced, which are essential for cognition. In humans, the synapse density increases rapidly after birth and reaches significant levels by the age of 2–3 years (Huttenlocher, 1979, 1984; Huttenlocher et al., 1982; Huttenlocher and Dabholkar, 1997). In rodents, synaptic density peaks during the second week after birth and reaches the adult level by week 3–4 of age (Crain et al., 1973; Semple et al., 2013). The timing of synapse peak and elimination is region specific. The increase in synaptic density is correlated with the increase of N-Methyl-D-Aspartic Acid Receptors (NMDARs) density in the cortex which peaks at the age of 1–22 years in humans and PND28 in rats (McDonald et al., 1990; Zhong et al., 1995; Zhang, 2006). Interestingly, rodent studies have shown that sleep is associated with an overall loss of cortical spine while a net gain of the spine was observed to increase during wakefulness (Maret et al., 2011). Furthermore, in a longitudinal study of sleep EEG conducted during childhood and adolescence periods (Campbell and Feinberg, 2009), found a steep decline of NREM delta and theta waves during the adolescent stage (11–16.5 years). The authors of the study suggested that EEG changes probably are reflective of synaptic pruning indicated by a reduction in cortical thickness as measured by MRI methodology in adolescent subjects (Shaw et al., 2008). In a cross-sectional study including children and adolescents the topography of SWA was highest over the posterior brain region in children, which then shifted to the frontal cortex in adolescents. This shift matches with the course of gray matter maturation (Kurth et al., 2010a; Smith et al., 2015). The fact that SWA amplitude increases during childhood and reaches maximum levels during puberty, and then decreases during adolescence, suggests the interesting possibility that SWA is probably the driving mechanism for brain maturation processes (Ringli and Huber, 2011).

The studies which have measured the cortical volume and the synaptic density across development in humans and rats have established a correlation between SWA coherence and cortical maturation (Kurth et al., 2015). This proposed role of SWA in brain maturation is further strengthened by the synaptic homeostasis hypothesis dsicussed above. It is reasonable to suggest that SWA is an indicator of synaptic density and synaptic strength during the process of development (Ringli and Huber, 2011). Furthermore, cortical maturation is assocaited with progressive changes in behavioral and cognitive functions from childhood to adulthood, which relies on synaptic pruning and neuronal myelination (Luna and Sweeney, 2004), supporting the link between SWA, cortical maturation and development processes of behavior, cognition and other functions. Considering all the important changes that occur within the brain during the developmental processes of early life, this period seems highly sensitive to sleep deprivation. Yet the exact relationship between brain maturation, SWA and behavior is not well-understood and needs further investigation. This is important as several neurobehavioral disorders are associated with sleep problems, suggesting a bidirectional relationship between sleep and neurobehavioral disorders. Studies conducted in a variety of animal models especially the rodent models of sleep deprivation by various groups around the world have shed important light into the role sleep plays in regulation of neurobehavioral functions. Few models are discussed below.

## Signaling Molecules Impacted by Sleep Deprivation

Sleep deprivation has a profound impact on the molecular biology of the brain, altering numerous signaling pathways including hippocampal glutamate, acetylcholine, and GABA systems. Studies have shown that sleep deprivation can lead to metabolic and cognitive disturbances in brain areas involved in learning, memory, and emotion such as the hippocampus, amygdala, and the prefrontal cortex (Havekes et al., 2012). For instance, a study using *in vitro* electrophysiological recordings found that sleep deprivation negatively impacts hippocampal long-term potentiation (LTP) and related signaling molecules significantly in both the dentate gyrus and CA1 areas (Alhaider et al., 2010), which plays an important role in learning and memory. This is suggestive to be a product of disruptive changes in intracellular signaling molecules and receptors including NMDA and AMPA receptors.

Sleep can modulate excitatory synaptic transmission and NMDA receptor function in the hippocampus, which is a functional contributor to synaptic plasticity (Alkadhi et al., 2013). Studies have further shown that the function of NMDA receptors in the dentate gyrus and CA1 were disrupted after 24 h REM sleep deprivation in mice (Chen et al., 2006).

Sleep deprivation can also negatively impact other molecular signaling pathways such as the cAMP/PKA pathway by increasing levels of phosphodiesterase IV, which decreases the levels of cAMP and impairs LTP and memory (Vecsey et al., 2009). Regulation of GABA receptors is also essential for memory consolidation by maintaining the correct level of synaptic inhibition. Studies suggest that sleep deprivation might increase GABAergic signaling and suppress activity of excitatory neurons (Havekes et al., 2012). Furthermore, synaptic plasticity is associated with increased expression of many other proteins such as presynaptic synapsin 1 and postsynaptic density protein (PSD95), in addition to dendritic CaMKII and microtubule-associated protein 2 (MAP2) (Kornau et al., 1995; Roberts et al., 1998; Sato et al., 2000). Interestingly, expression of PSD95 and NMDA receptors showed a similar pattern and GluN2B subunits co-localized with PSD95 in cultured rat hippocampal neurons, suggesting the importance of this interaction on synaptic plasticity at excitatory synapses (Kornau et al., 1995). Synaptic plasticity is also regulated by many other factors such as the neuronal growth factors and downstream signaling pathways. The brain-derived neurotrophic factor (BDNF), and the cyclic AMP response element binding protein (CREB) are among the factors that play an important role in synaptic plasticity (Figurov et al., 1996).

## Animal Models of Sleep Deprivation

To elucidate the cellular and molecular basis of sleep deprivation associated detrimental effects, several methods have been developed to induce sleep deprivation in animals, mainly in rodents. Some of these methods and the advantages/disadvantages of these methods and the mechanistic understanding obtained from these studies are discussed below.

### Platform Over Water

This method selectively deprives rodents of REM sleep. In this method, the rodents are placed in a tank with single or multiple platforms surrounded by water. The size of the platforms allows the animals to sleep on it, but once they lose their muscle tone as they enter REM sleep, they fall in the water and are awakened, hence preventing them from entering REM sleep (Jouvet et al., 1964). The control animals placed in a similar chamber with larger platforms that allow the animals to sleep. However, control animals in this method have been reported to show some alterations in behavioral and neuronal functions, suggesting the involvement of factors other than REM sleep deprivation as being responsible for some of the phenotypes (Marks and Wayner, 2005).

### Forced Locomotion

The animals in this method are placed individually in a chamber with a rotating drum or revolving floor which forces the animals to keep moving. This method induces total SD; it can also be modified to selectively target one sleep stage by continuous EEG monitoring (Friedman et al., 1979; Roman et al., 2005). However, the forced activity may cause fatigue for the animals inducing external stress, which has the potential to mask the effect of SD (Havekes et al., 2012; Alkadhi et al., 2013).

### Gentle Handling

The animals in this method are disturbed by the gentle shaking of their home cages or continuous introduction of new objects or nesting materials (Alkadhi et al., 2013). This method is very effective in inducing total sleep deprivation. However, this method involves constant vigilance by the investigator which makes it suitable only for short periods of SD. Moreover, personnel involvement and the introduction of new objects or nesting materials might present as additional confounding factors (Havekes et al., 2012; Alkadhi et al., 2013).

### Pinnacle Automated SD System

This apparatus consists of a cylindrical cage with a rotating bar at the base, while the bar is rotating, it gently touches the animal's feet keeping them awake. This system effectively produces sleep deprivation in rats as validated by polysomnography (Hines et al., 2013; Wooden et al., 2014). This system utilizes an automated rotating bar which gently pushes the pups to move in a timed manner. the rotating bar is not only gently and constantly disrupts the sleep of pups but also eliminates personnel involvement. Furthermore, in this model, two littermate pups can be placed in the sleep deprivation (SD) apparatus together. Having the littermate pups together during SD protocol is a significant procedural advantage as this further eliminates the concern of adding isolation stress (Atrooz et al., 2019; Atrooz and Salim, 2020). A comparison of some sleep deprivation models highlighting the major findings of the studies with regards to the mechanistic underpinnings of sleep deprivation and neurobehavioral functions are summarized in Table 1.

**Table 1:** Selected sleep deprivation models and key findings.

**Sleep deprivation method**	**Description**	**Main findings**	**References**
Modified Multiple Platform (MMP)	Mice (3xTgAD) were subjected to daily mild sleep restriction for 6 h/day for 6 weeks using the MMP technique.	a) Worsening of memory loss, and an accentuation of the accumulation of pTau and Aβ in the cerebral cortex. b) Significant positive correlations between plasma corticosterone levels and pTau and Aβ pathologies.	Rothman et al., 2013
Automated cage shaking stimulus	The animals were sleep deprived for 48 h in an automated cage shaking SD apparatus. SD stimulus such as sound, light, and vibration was provided.	a) Sleep loss affects the immune functions and increases complement C3 level in the hippocampus and is suggested to be associated with sleep loss. b) This study showed increased levels of complement units along with increased expression of C3aR and C5aR in sleep-deprived rats. c) Sleep deprivation (SD) memory impairment and neurogenesis decline via complement activation.	Wadhwa et al., 2019
	A microglia-mediated chronic neuroinflammatory model was proposed, which suggested that accumulated Aβ deposits and exocytosed tau bind toll-like receptors (TLR) on microglia in the brain in the developmental phase of AD.	a) Sleep deprivation (SD) and circadian rhythm disruption (CRD) is likely to be associated with a positive risk in developing Alzheimer's disease in humans. b) SD may potentially induce neuroinflammation via chronic microglial activation and systemic inflammatory response, aggravating AD progression.	Wu et al., 2019
Handling method	C57BL/6J (B6J) male mice were sleep deprived for 2, 4, and 6 h from the onset of the light phase by gently touching the cages when they started to recline and lower their heads.	A splicing mutation of the *Sik3* protein kinase gene causes a profound decrease in total wake time, due to an increase in inherent sleep need. SIK3 functions in the intracellular signaling pathway that dictates sleep need and regulates the daily amount of sleep.	Funato et al., 2016
Flower pot model	SD was induced using the flower pot technique for 96 h. Male Wistar rats (150–200 g, 6 weeks old) were individually housed on a narrow circle platform (6 cm in diameter and 2 cm in height) placed into a standard cage (43 × 22 × 21 cm^3^) surrounded by 1 cm of water. Control rats (vehicle-treated controls, melatonin-treated control) remained in their home cages (four rats per cage) during the experiment.	a) SD induces oxidative stress through glial activation and decreases fragile X-mental retardation protein (FMRP) expression in neurons. b) The efficacy of melatonin for the treatment of sleep-related neuronal dysfunction, which occurs in neurological disorders such as Alzheimer's disease and autism. c) Sleep deprivation leads to cognitive decline. d) Sleep deprivation induces glial activation and oxidative stress. e) Sleep deprivation alters the balance of FMRP expression in the brain. f) Melatonin prevents neuronal cell death and ROS production through FMRP regulation	Kwon et al., 2015
Automated cage shaking stimulus	Animals (adult male Sprague–Dawley rats (250–300 g) were sleep deprived for 48 h. They were kept single in transparent acrylic cages (35 × 35 × 60 cm, top open).	a) 48 h SD impaired novel object as well as location recognition memory along with reduced presynaptic (synaptophysin & synapsin I) and postsynaptic (PSD-95) protein expression in the hippocampus. b) Caffeine and modafinil treatments each rescued the changes in presynaptic and postsynaptic proteins and improved recognition behavioral deficits during SD.	Wadhwa et al., 2015

## Conclusion

It has become evident from a variety of animal studies that many neurobehavioral impairments develop upon sleep deprivation. This is particularly problematic when persistent sleep disruption happens during the critical time of brain development, as key processes such as cortical maturation and synaptic pruning may be hampered from lack of sleep. Exactly how and why that happens is an area that needs greater inquiry which will likely reveal circuit level information. As discussed earlier, NMDA, AMPA, cAMP-PKA and GABAergic pathways by involving critical signaling molecules such as PSD95, CREB, BDNF, GluN2B, CAMKII, CAMKIV, calcineurin etc. are plausible attractive molecular targets for effective therapeutic interventions.

## Data Availability Statement

The raw data supporting the conclusions of this article will be made available by the authors, without undue reservation.

## Author Contributions

GA, AH, and AP conducted literature review and prepared the first draft of this review article. SS and FA revised the first draft of the manuscript. SS finalized the draft after several layers of edits and iterations by all authors. All authors contributed to the article and approved the submitted version.

## Funding

Funding for this research was provided by a grant from the National Institutes of Health (2R15 MH093918-02), GEAR, and SGP grants awarded to SS.

## Conflict of Interest

The authors declare that the research was conducted in the absence of any commercial or financial relationships that could be construed as a potential conflict of interest.

## Publisher's Note

All claims expressed in this article are solely those of the authors and do not necessarily represent those of their affiliated organizations, or those of the publisher, the editors and the reviewers. Any product that may be evaluated in this article, or claim that may be made by its manufacturer, is not guaranteed or endorsed by the publisher.
